# Integrated transcriptomics and metabolomics provides insights into the *Nicotiana tabacum* response to heat stress

**DOI:** 10.3389/fpls.2024.1425944

**Published:** 2024-07-22

**Authors:** Hao Chen, Shaoxin Qiu, Yuanping Chen, Jiqin Li, Tingyu Xu, Pingzhan Zhong, Xiuhong Shao, Shihuan Xu, Zhuwen Ma, Zhenrui Huang, Xiaoying Pan

**Affiliations:** ^1^ Guangdong Key Laboratory for Crops Genetic Improvement, Crops Research Institute, Guangdong Academy of Agricultural Sciences (GAAS), Guangdong Provincial Engineering & Technology Research Center for Tobacco Breeding and Comprehensive Utilization, Guangzhou, China; ^2^ College of Agronomy, South China Agricultural University, Guangzhou, China; ^3^ China National Tobacco Corporation, Guangdong Company, Guangzhou, China

**Keywords:** tobacco, heat stress, transcriptome, metabolome, sugar metabolism, purine metabolism

## Abstract

Heat stress is a prevalent factor that significantly damages crops, especially with the ongoing global warming and increasing frequency of extreme weather events. Tobacco is particularly sensitive to temperature fluctuations, experiencing reduced yield and quality under high temperatures. However, the underlying molecular mechanisms of heat resistance in tobacco remain poorly understood. This study comprehensively analyzed biochemical, transcriptomic, and metabolomic responses to heat stress on the root and shoot of the tobacco cultivar K326 compared to control conditions. Heat stress significantly increased the activities of antioxidant enzymes (CAT, POD, and SOD) and levels of osmotic mediators (soluble sugars, sucrose, and proline) in the shoot. Furthermore, transcriptome analysis identified 13,176 differentially expressed genes (DEGs) in the root (6,129 up-regulated and 7,047 down-regulated) and 12,283 DEGs (6,621 up-regulated and 5,662 down-regulated) in the shoot. The root had 24 enriched KEGG pathways, including phenylpropanoid metabolism, while the shoot had 32 significant pathways, such as galactose metabolism and MAPK signaling. The metabolomic data identified 647 metabolites in the root and 932 in the shoot, with carbohydrates and amino acids being the main categories. The root had 116 differentially abundant metabolites (DAMs) (107 up-regulated and 9 down-regulated), and the shoot contained 256 DAMs (251 up-regulated and 5 down-regulated). Joint transcriptome and metabolome analysis showed that galactose metabolism and starch and sucrose metabolism were co-enriched in both tissues. In contrast, amino sugar and nucleotide sugar metabolism was enriched in the root, and purine metabolism in the shoot. The purine metabolic pathway in the shoot can modulate the expression of MYB transcription factors by influencing ABA synthesis and signaling, thereby controlling the accumulation of HSPs, raffinose, sucrose, and trehalose to enhance heat tolerance. Furthermore, *NtMYB78*, an MYB transcription factor, enhances tolerance for heat stress in tobacco. This research offers a foundational framework for investigating and implementing heat-resistant genes and metabolic pathways in the root and shoot of tobacco seedlings.

## Introduction

The escalating phenomenon of global warming has led to exceptionally high temperatures, significantly impacting crop productivity and diversity. Research suggests that every 1-degree Celsius increase in the global mean temperature could reduce the yields of wheat, rice, maize, and soybean by 6.0, 3.2, 7.4, and 3.1%, respectively, substantially threatening global food security (Shekhawat et al., 2022). High temperatures significantly reduce the yield and quality of tobacco, a plant species sensitive to temperature fluctuations (Yang et al., 2018). As a vital economic crop contributing to national tax revenue, the cultivation and utilization of heat-resistant tobacco varieties is paramount (Lu et al., 2011). Therefore, investigating the molecular mechanisms underlying heat resistance in tobacco is an important foundational framework for future breeding efforts.

The cell membrane system is the primary defense mechanism against heat stress and facilitates the transmission of heat-responsive signals. Thus, elevated temperatures can impair cell membrane integrity, heightening membrane permeability, compromising thermal stability and osmoregulation, and ultimately causing cellular demise (Mathur et al., 2014; de Pinto et al., 2015; Fortunato et al., 2023; Saeed et al., 2023). High temperatures cause an imbalance in reactive oxygen species in plant cells, leading to the accumulation of harmful substances like hydrogen peroxide and malondialdehyde, putting plants at risk of oxidative damage (Xie et al., 2019; Zhao et al., 2021; Sato et al., 2024). Plants have developed intricate heat stress response mechanisms, such as pathways for temperature sensing, signaling, gene expression regulation, protein synthesis, and metabolic regulation, in order to alleviate the detrimental effects of elevated temperatures (Ohama et al., 2017; Guihur et al., 2022; Shekhawat et al., 2022; Kan et al., 2023). When plants are subjected to high temperature stress, they detect stimuli through receptors located on the cell membrane and and subsequently relay these signals to the nucleus. Signaling molecules such as Ca^2+^ and MAPKs (mitogen-activated protein kinases) play key roles in heat stress signaling (Niu et al., 2020; Haider et al., 2021). The phytohormone ABA is important in response to heat stress. Overexpressing the ABA-responsive element binding protein (AREB) can improve heat tolerance in *Arabidopsis* (Suzuki et al., 2016). Moreover, ABA regulates *ZmCDPK* to improve ROS clearing and adapt to high-temperature environments (Zhao et al., 2021). Furthermore, MYB transcription factors are significant in response to stress, particularly abiotic stresses such as drought, temperature fluctuations, and salinity (Li et al., 2015; Jacob et al., 2021; Wang et al., 2021b). Heat stress transcription factors (HSFs) are essential regulators in plants that are activated in response to heat stress, binding to heat stress elements (HSEs) to stimulate the expression of heat stress proteins (HSPs) (Andrási et al., 2021; Tan et al., 2023; Wen et al., 2023). HSPs, a group of molecular chaperones, facilitate proper protein folding and prevent aggregation, playing a vital role in plant defense mechanisms against heat stress (Jacob et al., 2017; Song et al., 2021; Mondal et al., 2023).

Plant sugars serve multiple functions as energy sources, antioxidants, and essential components of cellular structures. Additionally, these sugars are crucial in regulating plant growth and development and aiding in plant adaptation to challenging environments (Chng et al., 2014; Proels and Hückelhoven, 2014). Monosaccharides such as hexose glucose, fructose, and certain glucose derivatives and disaccharides sucrose and trehalose-6-phosphate regulate various biological processes (Halford et al., 2003; Nunes et al., 2013; Tognetti et al., 2013). For instance, salinity and drought stress elevate the sucrose levels in the phloem sap of Arabidopsis, thereby maintaining the water potential (Durand et al., 2016). Trehalose interacts with various sugars, osmoprotectants, amino acids, and phytohormones to modulate metabolic reprogramming, which is essential for adaptation to heat stress (Luo et al., 2018; Zhang et al., 2022; Raza et al., 2023).

Thus, analyzing the transcriptomes and metabolomes can unravel key plant metabolic pathways and regulatory networks, improving the understanding of stress resistance in crops as sequencing costs decrease (Wang et al., 2019; Zhao et al., 2019). Many studies have used single or combined transcriptome and metabolome analyses to study cold and drought tolerance in tobacco (Jin et al., 2017; Luo et al., 2022b; Hu et al., 2023), and few on heat stress. Therefore, this study utilized an integrative analysis to investigate the transcriptome and metabolome of the root and shoot tissues of K326 tobacco. The main co-enriched pathways in both tissues were starch and sucrose metabolism and galactose metabolism. The amino acid sugar metabolism pathway was specifically and primarily enriched in the root, and purine metabolism was enriched in the shoot. Heat stress up-regulated key genes involved in sugar metabolism, including *raffinose synthase* (*RS*), *sucrose synthase* (*SUS*), *trehalose 6-phosphate synthase* (*TPS*), and *trehalose 6-phosphate phosphatase* (*TPP*), significantly increasing the levels of raffinose, sucrose, and trehalose. The MYB transcription factor, *NtMYB78*, discovered in this study, plays a crucial role in enhancing the heat stress response in tobacco plants. Our research can serve as a foundation for identifying key genes involved in the heat stress response in tobacco.

## Materials and methods

### Plant growth and heat treatment

The seeds of cultivated tobacco (*Nicotiana tabacum* L.) K326 (used in this study) were treated with 75% alcohol at room temperature for 5 minutes and washed five times with sterile water. Subsequently, the seeds were planted in small plugs and allowed to grow until they developed two small leaves before transplantation. At the five-leaf stage, eight similar-sized tobacco plants were heat treated at 45°C, 50% relative humidity, 16 h light/8 h dark photoperiod, and 10000 lux light intensity for 24 h. The control group was subjected to identical light and humidity conditions at 25°C. The root sample consists of all the root systems, while the leaves and stems of each plant were combined into one shoot sample. The root and shoot were collected for transcriptome sequencing.

### Physiological and biochemical assays

#### Relative conductivity assays

Here, the leaves were washed with distilled water and dried, cut into long strips (avoiding the main veins). Next, three fresh samples (0.1 g each) were placed in a centrifuge tube containing 10 mL of deionized water, and their conductivity before boiling (R1 and R2) was determined using a DDS-307 conductivity meter. The relative conductivity was calculated as R1/R2×100%.

#### Enzyme activity assays

Here, 0.2 g of fresh leaves were ground into powder using liquid nitrogen and 2 mL pre-cooled PBS (50 mM pH 7.8). The mixture was homogenized in an ice bath, transferred to a centrifuge tube, washed with PBS, and centrifuged at 8000 rpm for 15 min at 4°C. The supernatant was identified as the crude enzyme solution, and the levels of malondialdehyde (MDA) and enzymatic activities (catalase (CAT), superoxide dismutase (SOD), and peroxidase (POD)) were assessed following prior protocols (Hu et al., 2016).

#### Total soluble sugar and sucrose assays

Soluble sugars were quantified using anthrone colorimetry as outlined in a previous study (Abdel-Basset et al., 2010). The sucrose content was quantified using the plant sucrose content assay kit (Shanghai Acmec Biochemical Co. Ltd, Shanghai, China), following the guidelines outlined in the instruction manual.

#### Proline assays

Here, 0.5 g of leaves were ground and placed in a test tube containing 5 mL of 3% sulfosalicylic acid solution. The mixture was heated in a boiling water bath for 15 minutes, filtered, and filtrate collected. Next, 2 mL of the extract was combined with equal amounts of acetic acid and ninhydrin in a new test tube and sealed. The sealed test tube was heated in boiling water for 15 minutes, then cooled, and the solution was mixed with 5 mL toluene for extraction. The toluene layer was transferred to a cuvette, and its absorbance was measured at 520 nm using a spectrophotometer (Rajametov et al., 2021).

### RNA-seq library preparation and data analysis

Total RNA was isolated from tobacco root and shoot tissues using Trizol Reagent (Thermo Fisher Scientific, MA, USA). The RNA quality was evaluated using the Agilent 2100 Bioanalyzer (Agilent Technologies, CA, USA). Approximately 1 µg of RNA was utilized as input material for the transcriptome library preparation per sample. The libraries underwent sequencing on an Illumina NovaSeq platform (Tianjin, China), resulting in the generation of 150-bp paired-end reads.

The raw data was quality controlled using the FastQC software, eliminating reads containing adapter sequences, N (representing undetermined bases), and those with low-quality (> 50% of bases having a quality score < 5). Additionally, the Q20, Q30, and GC content were calculated for the clean data. Next, HISAT2 (v2.0.5), the reference genome index, was constructed with Nitab v1.0 Chr Edwards 2017 as the reference genome. The paired-end clean reads were aligned to the reference genome using HISAT2 v2.0.5, and FeatureCounts (v1.6.4) was employed to count the reads mapped to each gene (Liao et al., 2014; Kim et al., 2019). Subsequently, the FPKM (Fragments Per Kilobase of transcript per Million mapped reads) for each gene was computed based on gene length. The Pearson correlation coefficients among various samples were computed utilizing the cor function within the R package, and subsequently visualized as a heatmap through the pheatmap software. DESeq2 software (1.20.0) was utilized for differential gene expression analysis between the two comparison groups. Genes with expression levels of |log2 (fold change)| ≥ 1 and p-value ≤ 0.05 were retained for further analyses. The DEGs were further subjected to GO and KEGG enrichment analysis using clusterProfiler software (3.8.1) (Yu et al., 2012).

### Metabolite extraction and data analysis

A 100 mg tissue sample was flash-frozen in liquid nitrogen, transferred to an Eppendorf tube, and mixed with 500 μL of 80% methanol solution. The samples were then subjected to a 5-minute incubation on ice, followed by centrifugation at 15,000 g and 4°C for 20 minutes. One volume of the supernatant was extracted and diluted with mass spectrometry-grade water to a final methanol content of 53%. The specimen was centrifuged again at 15,000 g and 4°C for 20 minutes, then the supernatant was collected for subsequent analysis via liquid chromatography-mass spectrometry (LC-MS/MS).

The targeted metabolomics investigation (Dunn et al., 2011) was performed on the QTRAP^®^ 6500+ mass spectrometry platform (SCIEX, MA, USA). The detailed parameter settings of the instrument were referred to previous studies (Wang et al., 2021a).

The identified metabolites were characterized on the KEGG (https://www.genome.jp/kegg/pathway.html), HMDB (https://hmdb.ca/metabolites), and LIPIDMaps (http://www.lipidmaps.org/) databases. Principal components analysis (PCA) was conducted using the metaX software. T-test was used to determine the statistical significance (P-value) and fold change (FC) of metabolites between two groups. Metabolites meeting the criteria of a < 0.05 p-value, > 1 VIP (Variable Importance in Projection), and |log_2_fold change|≥1 were considered as differentially expressed. A volcano plot displaying the differentially abundant metabolites (DAMs) was generated using the ggplot2 package in R. Subsequently, Pearson correlation analysis was conducted on the DAMs using the cor () function in R. The statistical significance was determined using the cor.mtest () function in R, with a *p*-value of < 0.05 indicating statistical significance. The corrplot package in R was employed to visualize the correlation matrix, and a bubble plot was generated using the ggplot2 package in R. The functions and metabolic pathways of the metabolites were explored using the KEGG database. Pathway enrichment was determined by comparing the x/n > y/n ratio, and pathways with *p*-value < 0.05 were considered as significantly enriched.

### Mapping the network of correlations between DEGs and DAMs

Pearson’s correlation coefficients were calculated using Cor in R (Version 4.2.3) to assess the relationship between genes and metabolites within each common pathway. Next, a network diagram was created using Cytoscape (Version 3.10.1) to show the metabolite-gene using Pearson’s correlation coefficients above 0.9 in all groups.

### Quantitative real-time polymerase chain reaction

High-quality RNA was extracted and reverse transcribed with the HiScript II 1st Strand cDNA Synthesis Kit (Vazyme, Nanjing, China). The gene-specific primers were designed using the Primer5.0 software, and qRT-PCR was performed using the CFX96 real-time PCR detection system (Bio-Rad, CA, USA). The 10 μL reaction included 1 μL cDNA template, 0.25 μL of each primer, 5 μL SYBR Green Mix, and 3.5 μL ddH_2_O. The thermal cycling program was performed as follows: 95°C for 30 s, 40 cycles of 95°C for 5 s, and 60°C for 30 s. Relative gene expression levels were calculated using the cycling threshold (Ct) 2^–ΔΔCt^ method with *NtACTIN* as the internal reference gene. Each group had three biological and three technical replicates. The information of genes for qRT-PCR analysis is listed in **Supplementary Table 1**.

### Virus-induced gene silencing of the target genes

Based on the online website VIGS tool (https://vigs.solgenomics.net/), specific primers were designed to amplify a 300 bp silencing fragment using the tobacco seedling root cDNA as a template, and the amplified fragment was ligated into the pCE2 TA/Blunt-Zero Vector (Vazyme Biotech, Nanjing, China) to obtain a positive clone by sequencing. The designed homology arm was cloned into the linearized pTRV2 vector using the homologous recombination method using *Eco*RI and *Kpn*I restriction endonucleases, and the positive plasmid was obtained by sequencing. The pTRV1, pTRV2-empty vector, pTRV2-*PDS* (positive control), and pTRV2-*NtMYB78* plasmids were transformed into Agrobacterium tumefaciens strain GV3010. Next, the positive single clones were picked out and cultured in 500 μL LB liquid medium containing 25 μg/mL rifampicin and 50 μg/mL kanamycin, with constant shaking at 200 rpm, 28°C overnight. The constructs were cultivated further for 16 to 24 hours at 28°C in 50 mL of the same LB medium with constant shaking until their OD600 reached 0.6-0.8. The mixture was centrifuged at 3000×g for 15 min, the supernatant was removed, and the OD was adjusted to 0.8-1 using the infection buffer (10 mM MES; 10 mM MgCl_2_; 200 μM AS). An Agrobacterium solution with equal concentration of pTRV1 and pTRV2-empty vector, pTRV2-*PDS*, and pTRV2-*NtMYB78* were separately mixed and left at room temperature for 2 hours. Syringes were used to inject 5-8 uniformly-growing 4-week-old tobacco (*Nicotiana benthamiana*) plants in each group. The seedlings were maintained at 18°C and darkness for 2 d. Then, they were cultured at 22°C/18°C, 16h day/8h night for approximately 10 d, when the pTRV2-*PDS* plants showed an albino phenotype. Samples were collected for RNA extraction and analyzed for *NtMYB78* expression. The information of primers was listed in **Supplementary Table 1**.

## Results

### Heat stress significantly altered the phenotype and physiological and biochemical indexes of K326 seedlings

**Figure 1:**
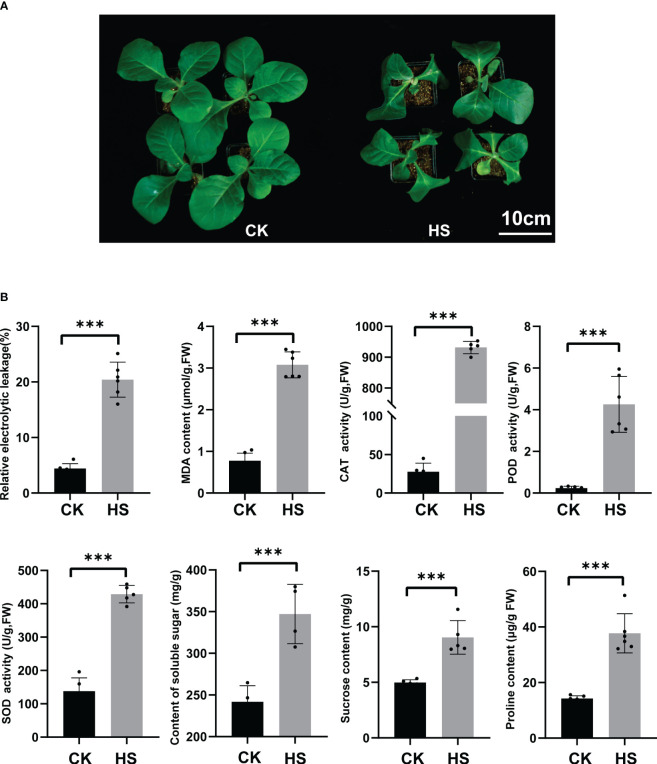
The phenotypic, physiological, and biochemical indicators of heat stress in tobacco seedlings. **(A)** The control group (25°C for 24h (CK left)), heat stress group (45°C for 24h. (HS, right)), Bar=10 cm. **(B)** Leaf relative conductivity, MDA content, CAT, POD, and SOD enzyme activity, soluble sugar content, sucrose content, and proline contents. Significance was assessed using more than four biological replicates, with asterisks denoting statistically significant differences between treatments as determined by the paired Student’s *t*-test (***P < 0.001).

After 24h of heat stress treatment at 45°C, the five-leaf stage K326 tobacco plants showed severe leaf wilting (**Figure 1A**). The notable rise in leaf relative conductivity following exposure to heat stress suggests a substantial impairment of leaf cell membrane integrity (**Figure 1B**). Moreover, heat treatment significantly elevated the content of MDA, the end product of lipid peroxidation in plant membranes and an important response parameter to the antioxidant capacity of the plant (Wang et al., 2016). Heat stress significantly increased the levels of antioxidant enzymes CAT, POD, and SOD, as did the concentrations of soluble sugar, sucrose, and proline (**Figure 1B**).

### Transcriptome analysis of tobacco seedlings in response to heat stress

Three biological replicates per tissue (collected at 0 and 24 hours) generated 112.13G of raw transcriptome sequencing data. Following quality control, a minimum of 7.89G clean data were obtained for each sample. The Q20 base percentage surpassed 98%, Q30 exceeded 96%, and the GC content was 42.56-44.94% (**Supplementary Table 2**). The three biological replicates of different tissues and time points were significantly correlated, with correlation coefficients exceeding 0.9 (**Supplementary Figure 1A**). Furthermore, the PCA results showed that the 12 samples were grouped into four categories, with similar biological replicates clustering based on tissue and treatment (**Supplementary Figure 1B**). The sequencing data were of high quality and consistent among replicates, making it suitable for further analysis.

Heat stress resulted in the up-regulation of 6129 genes and the down-regulation of 7047 genes in the root when compared to R24 and R0. In contrast, 6621 genes were up-regulated, and 5662 genes were down-regulated compared to S24 and S0 (**Supplementary Figures 2A, B**; **Supplementary Table 3**). Venn analysis revealed 2326 up-regulated and 2382 down-regulated genes shared between the root and shoot (**Supplementary Figures 2C, D**). More genes belonged to specific differentially expressed genes. It is implied that the root and shoot may have a specialized response mechanism to heat stress.

**Figure 2:**
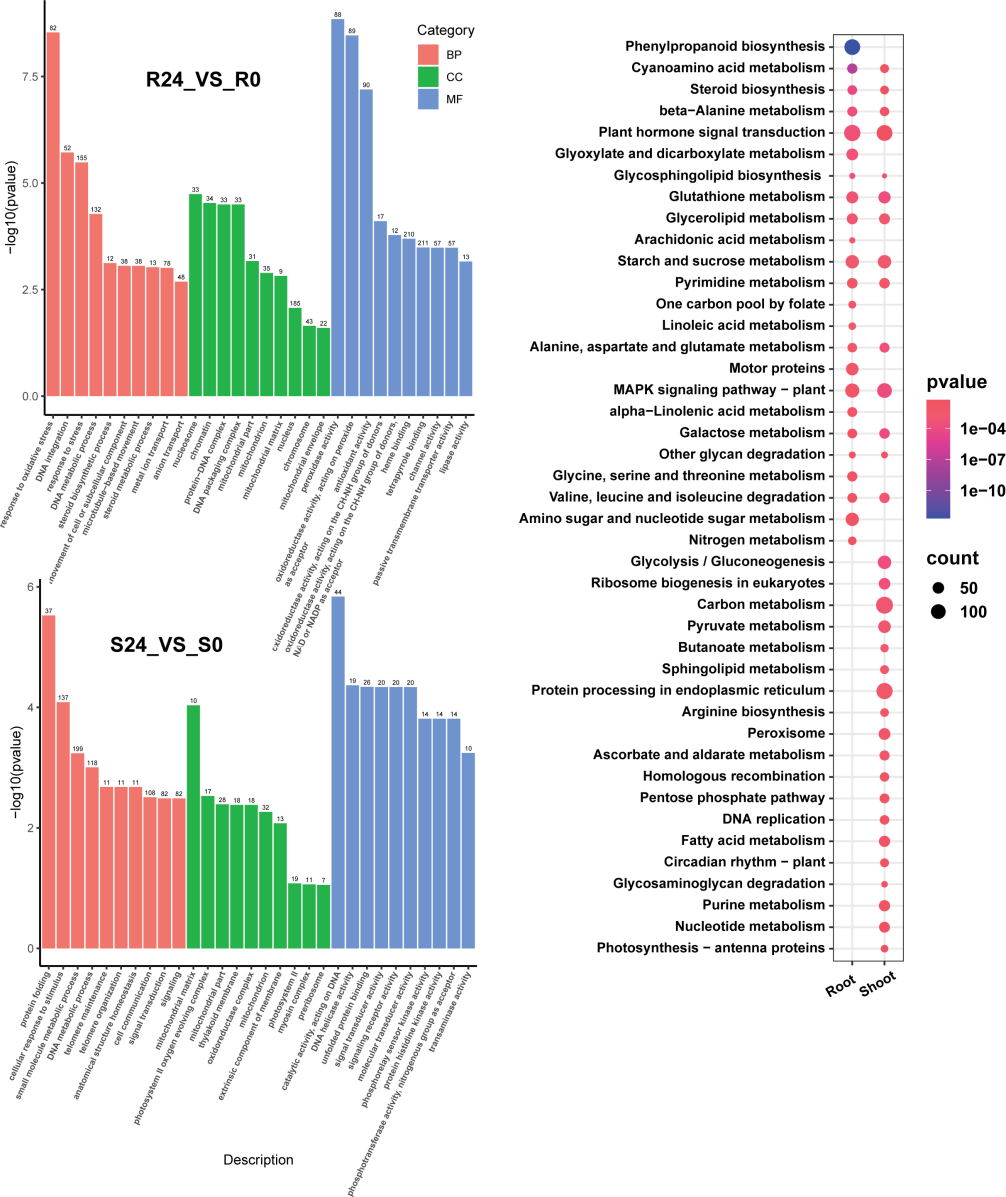
The GO and KEGG enrichment of the DEGs (before and after heat stress) in both the root and shoot. The top ten GO classifications under various stress conditions in each category include biological process (BP), molecular function (MF), and cellular component (CC) in the root tissue **(A)** and shoot **(B)**. **(C)** shows the enriched KEGG pathways in the root and shoot. The size of the circle indicates the number of genes, and the color indicates the enrichment significance (*p*-value).

The Gene Ontology (GO) analysis identified 103 significantly enriched GO terms in the root. The enriched Biological Processes (BP) predominantly encompassed stress response-related GO terms, including response to oxidative stress (GO:0006979), DNA integration (GO:0015074), response to stress (GO:0006950), and DNA metabolic process (GO:0006259). The significantly enriched Molecular Functions (MF) includes peroxidase activity (GO:0004601), oxidoreductase activity acting on peroxide as acceptor (GO:0016684), antioxidant activity (GO:0016209), and heme binding (GO:0020037). Finally, the significantly enriched Cellular Components (CC) comprised of the nucleosome (GO:0000786), chromatin (GO:0000785), protein-DNA complex (GO:0032993), and DNA packaging complex (GO:0044815) (**Figure 2A**; **Supplementary Table 4**).

A total of 110 GO terms were significantly enriched in the shoot tissue. The enriched BP category included protein folding (GO:0006457), cellular response to stimulus (GO:0051716), small molecule metabolic process (GO:0044281), and DNA metabolic process (GO:0006259). The enriched MF category included catalytic activity acting on DNA (GO:0140097), DNA helicase activity (GO:0003678), unfolded protein binding (GO:0051082), and signal transducer activity (GO:0004871). In the CC category, prominent terms comprised mitochondrial matrix (GO:0005759), photosystem II oxygen-evolving complex (GO:0009654), mitochondrial part (GO:0044429), and thylakoid membrane (GO:0042651). The GO terms for DNA metabolic processes were enriched in both tissues (under BP) and mitochondria (under CC). The GO terms enriched in the root predominantly pertain to antioxidant processes, including response to oxidative stress and stress (under BP), as well as peroxidase and antioxidant activities (under MF). In contrast, the GO terms enriched in shoot tissue are primarily associated with signaling responses and transduction, such as cellular response to stimuli and signal transduction (under BP), as well as signal transducer and signaling receptor activities (under MF) (**Figure 2B**; **Supplementary Table 4**).

A mapping analysis was conducted using the Kyoto Encyclopedia of Genes and Genomes (KEGG) database (http://genome.jp/kegg/) to examine the regulatory pathways of DEGs induced by heat stress. Heat stress enriched 126 pathways in the root, of which 24 were significantly enriched. The shoot had 126 enriched pathways, including 32 significantly enriched ones (**Figure 2C**; **Supplementary Table 5**). Half of the pathways significantly enriched in the root following heat stress were the same as those in the shoot, suggesting a common alteration in the metabolic pathways. These shared pathways include plant hormone signal transduction, glutathione metabolism, starch and sucrose metabolism, MAPK signaling pathway, and glucose metabolism pathway, which are crucial in plant stress response. Metabolic pathways that were notably enriched in the root consist of phenylpropanoid biosynthesis, amino sugar and nucleotide sugar metabolism, and nitrogen metabolism. Conversely, the pathways enriched in the shoot were specific to carbon metabolism, peroxisome, circadian rhythm-plant, and photosynthesis-antenna protein, all associated with the response to heat stress. These findings indicate that distinct adaptive mechanisms mitigate the effects of heat stress on various tissues.

### Transcription factors expressed in heat-stressed tobacco seedlings

**Figure 3:**
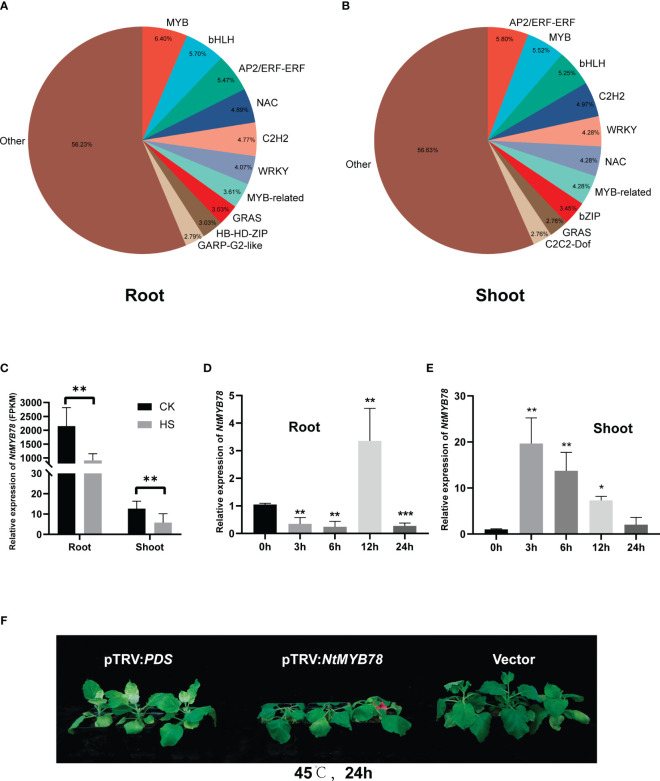
Distribution of transcriptome factors (TFs) in DEGs. **(A)** Statistical analysis of transcription factors in the root before and after heat treatment. **(B)** Statistical analysis of TFs in the shoot before and after heat treatment. **(C)** Expression of *NMYB78* in the RNA-seq data. The relative expression of *NtMYB78* in the root **(D)** and shoot **(E)** following heat treatments for 0, 3, 6, 12, and 24 hours. Significance analysis was conducted using a Student’s *t*-test with three biological replicates, each with a control treatment (0 hours), *P < 0.05; **P < 0.01; ***P < 0.001. **(F)** Phenotypic analysis of pTRV : *NtMYB78* plants after treatment at 45°C and 24h. pTRV-*PDS* was the positive control, and the vector was the negative control.

Transcription factors mediate plant responses to heat stress by regulating target gene expression. Thus, a statistical analysis of the diversity and abundance of TFs within the DEGs revealed 79 classes of TFs in the root, the top five classes being MYB (55), bHLH (49), AP2/ERF-ERF (47), NAC (42), and C2H2 (41). The shoot had 80 classes of transcription factors, and the top five most abundant ones included AP2/ERF-ERF (42), MYB (40), bHLH (38), C2H2 (36), and MYB-related (31). These TFs are involved in plant stress tolerance processes (Mizoi et al., 2012; Li et al., 2015; Guo et al., 2021) (**Figures 3A, B**; **Supplementary Table 6**).

Moreover, MYB and MYB-related transcription factors were the most prominent in the root and shoot, consistent with previous findings that MYB is crucial for enhancing plant stress tolerance (Li et al., 2019; Wang et al., 2021b). Therefore, we analyzed one of the MYB transcription factors, *Nitab4.5_0000895g0070*, which is homologous to *Arabidopsis thaliana AtMYB78*, hereafter referred to as *NtMYB78. NtMYB78* significantly decreased expression in both tissues after heat stress, with higher levels in the root than shoot (**Figure 3C**). Heat stress initially decreased *NtMYB78*, followed by an increase and peaking at 12 h before gradually declining in the root (**Figure 3D**). In the shoot, *NtMYB78* initially increased, peaking at 3 hours, followed by a decrease (**Figure 3E**). These findings suggest that the shoot responded more rapidly to heat stress than the root. To further verify whether *NtMYB78* was involved in heat stress response, we conducted VIGS-mediated knock-down of gene expression. The results revealed that *NtMYB78* expression level was significantly down-regulated in the pTRV : *NtMYB78* plants (**Supplementary Figure 3**). The pTRV : *NtMYB78* exhibited increased sensitivity following exposure to heat stress, suggesting a role for *NtMYB78* in the positive regulation of heat stress response (**Figure 3F**).

### Heat-responsive *HSPs* in heat-stressed tobacco seedlings

The heat shock proteins (HSPs), significant in the cellular response to heat stress (Tian et al., 2021; Kang et al., 2022; Mondal et al., 2023), were differentially expressed in tobacco roots and shoots under heat stress. In this study, heat stress differentially regulated 106 *HSPs* in the root, including *HSP20* (39), *HSP40* (12), *HSP70* (26), and *HSP90* (29). The shoot had 115 significant differentially expressed *HSPs*, including *HSP20* (37), *HSP40* (12), *HSP70* (31), and *HSP90* (36) (**Supplementary Figure 4A**; **Supplementary Table 6**). Using two genes from each HSP family (*HSP20*, *HSP40*, *HSP70*, and *HSP90*), qRT-PCR analysis revealed consistent expression with the transcriptome data (**Supplementary Figure 4B**).

### Differential metabolomes in heat-stressed tobacco seedlings

A comprehensive LC-MS/MS identified 647 metabolites in the root samples. The metabolites are predominantly grouped into categories of amino acids and their derivatives (21.51%), carbohydrates and their derivatives (12.02%), and lipids (11.57%) (**Supplementary Figure 5A**; **Supplementary Table 7**). The shoot contained 932 metabolites, including amino acids and their derivatives (19.31%), carbohydrates and their derivatives (10.41%), and organic acids, lipids, and their derivatives (collectively accounting for 10.19%) (**Supplementary Figure 5B**; **Supplementary Table 7**). The PCA of the root data revealed that PC1 accounted for 50.87% of the total variation, while PC2 explained 17.7% of the variation (**Supplementary Figure 5C**). The PCA of the shoot showed that PC1 and PC2 accounted for 51.91% and 15.42% of the total variation, respectively (**Supplementary Figure 5D**). Furthermore, the biological replicates within each group clustered in the PCA results, providing additional evidence of the group distinctions and indicating the reliability and suitability of the metabolite data for subsequent analysis.

**Figure 4:**
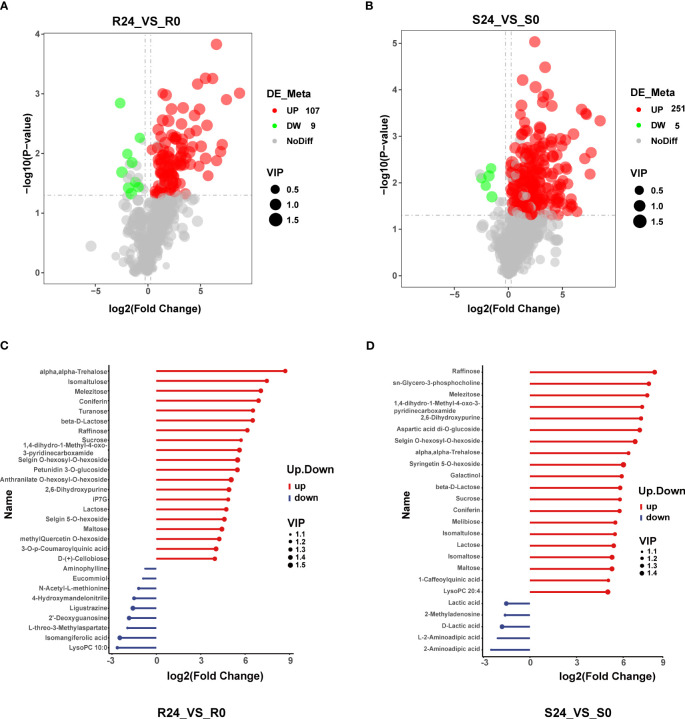
Differential metabolite analysis. **(A, B)** show a volcano map of DAMs in R24_VS_R0 and S24_VS_S0. Red dots indicate the up-regulation of DAMs, green dots indicate the down-regulation of DAMs, gray dots indicate no DAMs, and the circle size indicates the VIP value. **(C, D)** show the top 20 DAMs that increased and decreased in R24_VS_R0 and S24_VS_S0.

The root contained 116 DAMs (107 up-regulated and 9 down-regulated). The top 20 up-regulated compounds identified in the study included alpha-trehalose, isomaltulose, melezitose, coniferin, turanose, beta-D-Lactose, raffinose, sucrose, 1,4-dihydro-1-Methyl-4-oxo-3-pyridinecarboxamid, selgin O-hexosyl-O-hexoside, petunidin 3-O-glucoside, anthranilate O-hexosyl-O-hexoside, 2,6-Dihydroxypurine, iP7G, lactose, selgin 5-O-hexoside, maltose, methylQuercetin O-hexoside, 3-O-p-Coumaroylquinic acid, and D-(+)-cellobiose. Notably, sugar compounds accounted for most (11) up-regulated compounds. The nine most significantly down-regulated DAMs included LysoPC 10:0, isomangiferolic acid, L-threo-3-methylaspartate, 2’-deoxyguanosine, ligustrazine, 4-hydroxymandelonitrile, N-acetyl-L-methionine, eucommiol, and aminophylline. Terpenoids and amino acids were the most abundant classes, represented by two compounds (**Figures 4A, C**; **Supplementary Table 7**). The S24 vs S0 comparison group had 256 DAMs, including 251 up-regulated and 5 down-regulated compounds (**Figure 4B**). Further, the top twenty DAMs were raffinose, sn-glycero-3-phosphocholine, melezitose, 1,4-dihydro-1-methyl-4-oxo-3-pyridinecarboxamide, and 2,6-Dihydroxypurine, aspartic acid di-O-glucoside, selgin O-hexosyl-O-hexoside, alpha-trehalose, syringetin 5-O-hexoside, galactinol, beta-D-Lactose, sucrose, coniferin, melibiose, isomaltulose, lactose, isomaltose, maltose, 1-caffeoylquinic acid, and lysoPC 20:4. Notably, sugar compounds were the most abundant (12) among the top twenty DAMs. The top five down-regulated DAMs included 2-aminoadipic acid, L-2-aminoadipic acid, D-lactic acid, 2-methyladenosine, and lactic acid (**Figure 4D**). There were 51 co-up-regulated DAMs, carbohydrates and their derivatives being abundant (17), including lactose, raffinose, trehalose, sucrose, and sorbose. Amino acids and their derivatives were the second most abundant (10), including asparagine, histidine, nepsilon-acetyl-L-lysine, and proline (**Supplementary Figures 6A, C**). There were no shared and down-regulated DAMs (**Supplementary Figure 6B**). The shoot had significantly more DAMs than the root, suggesting substantial alterations in shoot metabolite following heat treatment. This disparity may be attributed to the heightened susceptibility of the shoot to heat stress relative to the root.

**Figure 5:**
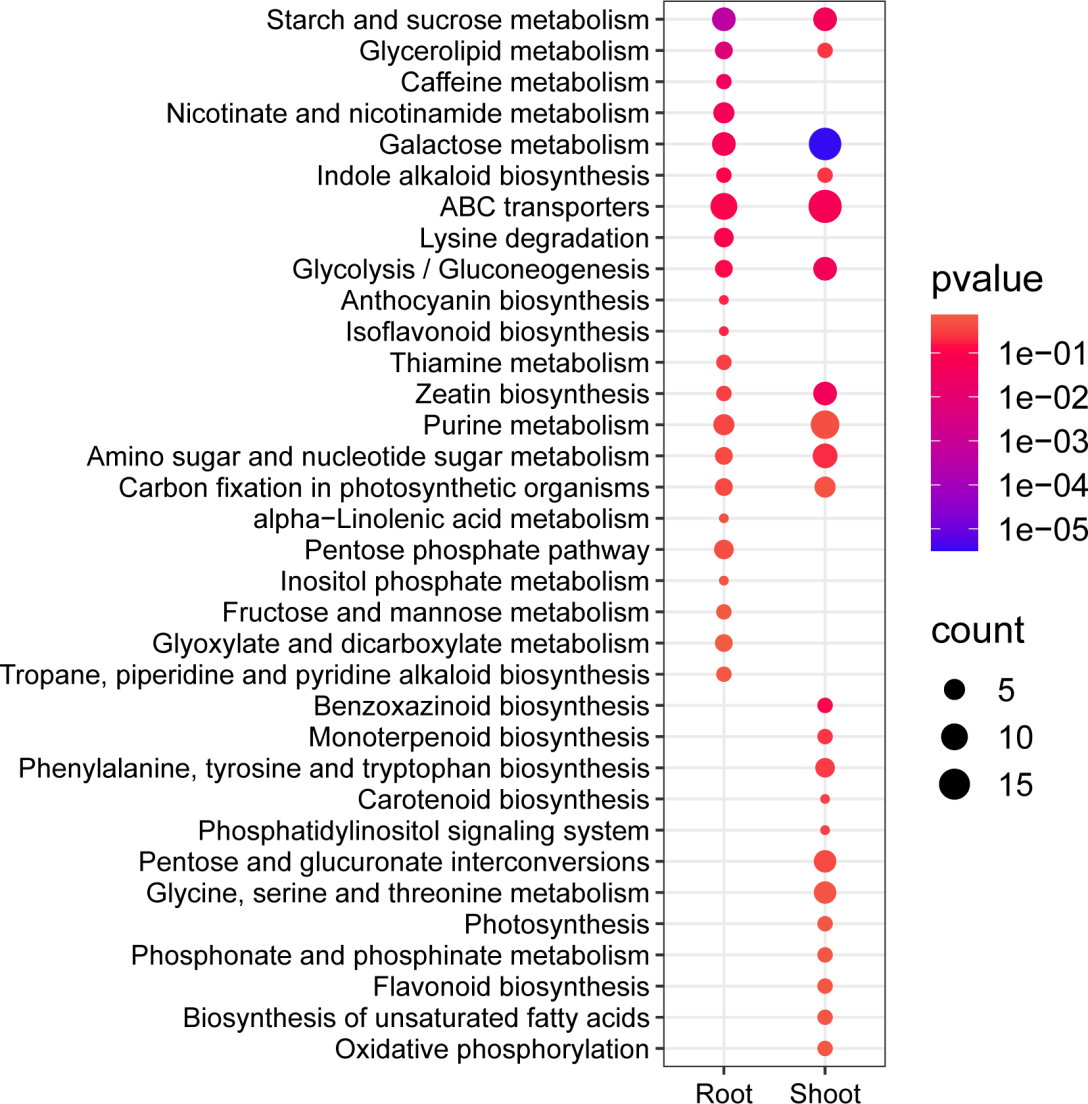
The differential metabolite KEGG enrichment analysis was conducted, with bubble plots illustrating the top 22 significantly enriched pathways in both the root and shoot tissues. The size of each bubble corresponds to the number of metabolites, while the color of the bubble represents the significance level as indicated by the *p*-value.

Furthermore, the root contained 33 significantly enriched KEGG pathways, including starch and sucrose metabolism, glycerolipid metabolism, and caffeine metabolism (**Figure 5**; **Supplementary Table 8**). The shoot DAMs enriched 41 pathways, the most significantly enriched being galactose metabolism, gluconeogenesis, starch and sucrose metabolism, and zeatin biosynthesis (**Figure 5**; **Supplementary Table 8**). The two tissues were co-enriched with various metabolic processes such as starch and sucrose metabolism, glycerolipid metabolism, galactose metabolism, ABC transporter, and purine metabolism. The pathways enriched in the root included caffeine metabolism, nicotinate and nicotinamide metabolism, lysine degradation, and the pentose phosphate pathway. In contrast, the shoot was primarily enriched with benzoxazinoid biosynthesis, phenylalanine, tyrosine, and tryptophan biosynthesis, pentose and glucuronate interconversions, and photosynthesis (**Figure 5**; **Supplementary Table 8**). In conclusion, shoots may demonstrate heightened complexity in their response mechanisms to heat stress.

### Integrated transcriptomics and metabolomics identified key heat-regulated metabolic pathways

**Figure 6:**
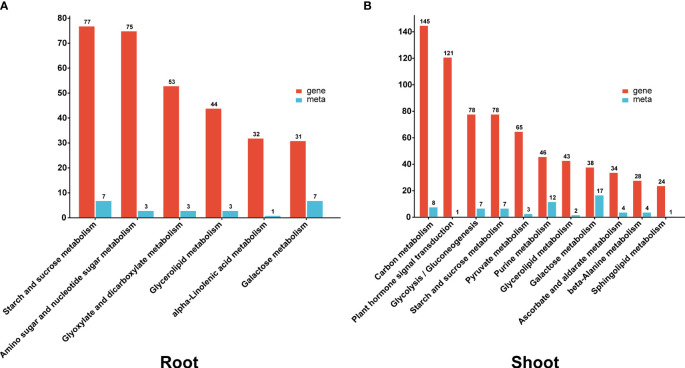
The **(A, B)** column diagrams show the KEGG co-enrichment pathway of DEGs (red column) and DAMs (blue column) in the root and shoot.

A combined analysis of the transcriptome and metabolites was performed on both the root and shoot tissues to further explore critical pathways of heat stress response. Heat stress regulated (DEGs and DAMs) and enriched several functional pathways in both tissues, including sucrose starch metabolism, glycerolipid metabolism, and galactose metabolism. Pathways for amino sugar and nucleotide sugar metabolism, glyoxylate and dicarboxylate metabolism, and alpha-linolenic acid metabolism were enriched exclusively in the root. In contrast, carbon metabolism, plant hormone signal transduction, pyruvate metabolism, and purine metabolism were specifically enriched in the shoot (**Figure 6**).

**Figure 7:**
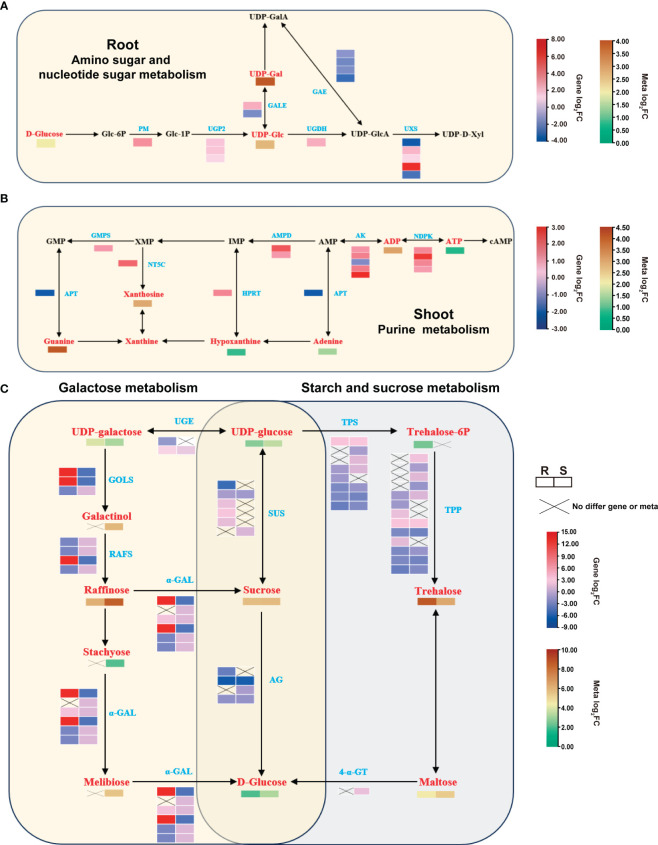
Major enriched metabolic pathways in the root and shoot after heat treatment. **(A)** DAMs and DEGs that significantly enriched the amino sugar and nucleotide sugar metabolism pathways in the root. **(B)** DAMs and DEGs that significantly enriched the purine metabolism pathway in the shoot. **(C)** DAMs and DEGs that co-enriched the galactose metabolism and starch and sucrose metabolism pathways in the root and shoot after heat treatment. Red represents DAMs, and blue represents DEGs.

Gene-metabolite correlation networks revealed a strong correlation (cor > 0.9 and *p*-value < 0.01) between the differential genes and metabolites in critical metabolic pathways. The root had major metabolic pathways, amino sugar and nucleotide sugar metabolism, enriched by 17 and 3 significantly correlated DEGs and DAMs, respectively. The corresponding enriched metabolic pathways included UDP-galactose, which is involved in the galactose metabolism pathway for response to heat stress (**Figure 7A**; **Supplementary Figure 7A**). Heat stress significantly enriched the purine metabolic pathway in the shoot, with 28 DEGs that were strongly correlated (predominantly displaying a positive correlation) to 11 DAMs. Heat treatment also generated DEGs and DAMs that enriched the purine metabolic pathway in the shoot (**Figure 7B**; **Supplementary Figure 7B**). Furthermore, heat stress up-regulated *adenine phosphoribosyltransferase* (*APT*), *adenylate kinase* (*AK*), and *nucleoside-diphosphate kinase* (*NDPK*) (**Supplementary Table 3**). Heat stress notably elevated the concentrations of guanine, xanthine, hypoxanthine, and adenine, pivotal intermediates in purine metabolism (**Supplementary Table 7**).

Heat stress significantly altered the abundance of sugars in both tissues. Specifically, the enriched galactose metabolic pathway in the root contained 12 DEGs, significantly associated with 7 DAMs. In the shoot, 24 DEGs were correlated with 15 DAMs (**Supplementary Figure 8A**). Similarly, the enriched sucrose starch metabolism pathway in the root contained 30 DEGs, which was significantly correlated with 7 DAMs, and the shoot had 38 DEGs, which was significantly associated with 7 DAMs (**Supplementary Figure 8B**). Sugar metabolism-related DEGs and DAMs were more abundant in the shoot than in the root. Subsequently, we integrated our RNA-seq and metabolomics datasets to contrust a gene-metabolite network (**Figure 7C**). The qRT-PCR results indicated a significant increase in the expression of *raffinose synthase* (*RS*), *sucrose synthase* (*SUS*), *trehalose 6-phosphate synthase* (*TPS*), and *trehalose 6-phosphate phosphatase* (*TPP*), consistent with the transcriptome data (**Supplementary Figure 8C**; **Supplementary Table 3**). Metabolomic analysis revealed significant increases in raffinose, sucrose, and trehalose contents in both root and shoot, with 52 - 419 fold changes (**Supplementary Table 7**). These results suggested that these genes may directly or indirectly regulate the accumulation of sugar metabolites in response to high-temperature stress.

## Discussion

### The shoot of tobacco seedlings are more sensitive to heat stress than the root

Extensive research has been conducted on the effects of heat stress on plant shoots; however, there remains a notable gap in the literature regarding the response of plant roots to heat stress (Wang et al., 2019; Liu et al., 2020; Xie et al., 2023). Thus, this study simultaneously exposed tobacco seedling roots and shoots to elevated temperatures to explore the comparative responses of roots and shoots to heat stress. Subsequent transcriptome analyses on the both tissues identified 13176 and 12283 differentially expressed genes (DEGs) in the root and shoot, respectively. The enriched GO terms in the root were associated with stress response and antioxidant pathways, whereas those in the shoot were predominantly related to plant signaling pathways. These findings are consistent with the GO-enriched terms observed in roots and leaves during a previous study on drought stress in switchgrass (Tiedge et al., 2022).

The root and shoot exhibited significant enrichment of KEGG pathways, with 24 pathways identified in the root and 32 in the shoot. More than half of the metabolic pathways were common to both tissues, including plant hormone signal transduction, MAPK signaling pathway, and starch and sucrose metabolism. The significant metabolic pathways enriched in response to heat stress align with previous reports on Arabidopsis and pepper (Wang et al., 2019; Olas et al., 2021). Heat stress mainly enriched phenylpropane metabolism, amino acid sugar and nucleic acid sugar metabolism in the roots. Phenylpropane metabolism is a key secondary pathway in plants, producing important metabolites like lignin, anthocyanins, and organic acids that regulate plant growth and stress response (Dong and Lin, 2021). Heat stress significantly enriched pathways related to carbon metabolism, purine metabolism, and peroxisomes in the shoot tissue. Furthermore, heat stress significantly elevated antioxidant enzyme activities in the shoot (**Figure 1B**), consistent with prior research (Silva et al., 2018).

The root and shoot had 647 and 932 metabolites, including 115 DAMs and 255 DAMs, respectively, suggesting that heat stress may affect the root less. There were 33 pathways identified for DAMs enrichment in the root and 41 pathways for DAMs enrichment in the shoot, indicating a greater complexity of metabolic pathways in the shoot response to heat stress.This result aligns with previous studies showing wheat shoots experience more metabolite changes during drought tolerance (Kang et al., 2019). The shoot exhibited a greater sensitivity to heat stress, as evidenced by more metabolite alterations and enriched KEGG pathways.

### Purine metabolism plays a key role in responding to heat stress

The integrated transcriptomic and metabolomic data indicated that the purine metabolism pathway was predominantly enriched in the shoot, and gene-metabolite correlations identified 28 and 11 significantly linked DEGs and DAMs. Therefore, purine metabolism may play a crucial role in the response to heat stress. Purines are necessary metabolites in all organisms since they regulate various cellular functions, such as cell signaling, redox metabolism, and energy metabolism (Kokina et al., 2019). Elevated temperatures notably increased purine metabolic compounds in the high-altitude fish *Triplophysa siluroides* (Chen et al., 2023). In quinoa, heat stress enriched the purine metabolic pathway and up-regulated substances related to purine metabolism in both heat-sensitive and heat-resistant varieties (Xie et al., 2023). The purine metabolic compounds adenine, xanthine, hypoxanthine, and guanine increased notably within the shoot (**Figure 7B**). A recent study found that cAMP, the first identified second messenger, influences heat stress response by regulating various cellular processes such as protein processing, ion balance, and the ubiquitin-proteasome system (Liang et al., 2022). Interestingly, the metabolome results demonstrated a significant elevation of ATP, which is a precursor substance of cAMP, and a 1.2-fold elevation of cAMP (**Supplementary Table 7**). It has been reported that the accumulation of allantoin, an intermediate product of purine metabolism, promotes ABA synthesis and participates in the response to adversity stress (Watanabe et al., 2014; Kaur et al., 2023). However, the content of allantoin in the shoot did not change significantly in this study, probably because of the early heat stress response. Consequently, this study analyzed genes associated with ABA synthesis and signaling, revealing a substantial up-regulation of *NCED1* (*Nitab4.5_0001924g0060*) (**Supplementary Figures 9A, C**). This pivotal gene implicated in ABA synthesis increased by 87-fold in the shoot expression, and ABA levels elevated by 8-fold (**Supplementary Tables 3**, **7**). Furthermore, heat stress increased the expression of the ABA-responsive element-binding protein (*ABF*), indicating their role in modulating the expression of ABA-responsive genes under heat stress (**Supplementary Figure 9B**; **Supplementary Table 3**).

MYB transcription factors play important roles in ABA signaling, and they can be regulated by ABA to influence plant responses to abiotic stresses (Ma et al., 2023; Du et al., 2024). These MYB transcription factors can also interact with MYB cis-elements in the promoters of target genes in response to abiotic stress (Park et al., 2011; Zheng et al., 2012; Wang et al., 2021b). Heat stress significantly altered 71 MYB and MYB-related transcription factors in the shoot (20 up-regulated and 51 down-regulated (**Supplementary Table 6**). *AtMYB78* in *Arabidopsis thaliana* is crucial for resistance to drought, salt, and oxidative stresses (Mengiste et al., 2003). The homologs of *AtMYB78* are equally significant in mediating responses to both biotic and abiotic stresses in wheat, tomato, and oilseed rape (Abuqamar et al., 2009; Liu et al., 2011; Chen et al., 2016). We therefore analyzed the expression profile of *NtMYB78*, exhibited a pattern of initial increase followed by decrease in both root and shoot tissues under varying durations of heat stress treatment. Notably, the shoot displayed a more rapid response, reaching peak expression levels after 3 hours of heat stress treatment (**Figures 3D, E**). This observation aligns with findings from a previous study on heat-stressed wheat, where MYB-associated transcription factors rapidly accumulated within one hour of heat stress (Zhao et al., 2017). Conversely, certain MYB transcription factors, such as *AtMYBS1*, have been shown to exert a negative regulatory effect on heat stress by inhibiting the expression of the MAX1 gene (Li et al., 2023). This was why more MYB transcription factors were down-regulated after 24 hours of heat stress.

MYB transcription factors can interact with HSFs to regulate HSP protein expression (Jacob et al., 2017; Priya et al., 2019). These HSPs are molecular chaperones for the assembly, stabilization, and maturation of proteins and protein complexes. They are also important in plant development and responses to abiotic and biotic stresses (ul Haq et al., 2019). The up-regulation of *BcHSP70* and *NtHSP70-8b* notably enhances heat tolerance in tobacco (Wang et al., 2016; Zhang et al., 2023). In this study, heat stress differentially expressed 106 (80 up-regulated, 26 down-regulated) and 115 (83 up-regulated, 32 down-regulated) HSPs in the root and shoot, respectively (**Supplementary Figure 2**). Thus, increasing the expression of HSPs could improve heat tolerance tobacco.

### Sugar metabolism pathways have a significant impact on heat tolerance

Sugars, including raffinose, trehalose, and sucrose, are osmoregulatory compounds that aid in plant adaptation to molecular, cellular, and physiological alterations (Sami et al., 2016; Raza et al., 2023). Specifically, UDP-galactose and myo-inositol use HSFA1 to modulate *GALACTITOL SYNTHASE* (*GOLS1*) expression, synthesizing galactitol. Galactitol produces raffinose using *RAFFINOSE SYNTHASE* (*RS*) in a temperature-dependent condition (Panikulangara et al., 2004; Cortijo et al., 2017). Therefore, heat-stressed plants improve heat tolerance by increasing the content of raffinose (Serrano et al., 2019; Yan et al., 2022). This study identified notable alterations in *GOLS* and *RS* expression levels in the root and shoot of heat-stressed tobacco. Specifically, heat stress significantly increased *RS* (*Nitab4.5_0002682g0060*) expression in both tissues by 6- and 9-fold, respectively. (**Figure 7C**; **Supplementary Table 3**). Metabolomic analyses further revealed a substantial 71- and 356-fold rise in the raffinose content in the root and shoot, respectively (**Figure 7C**; **Supplementary Table 7**). A recent study showed that trehalose-6-phosphate synthase 1 can regulate heat tolerance in *A. thaliana* by inducing raffinose accumulation (Reichelt et al., 2023). Trehalose biosynthesis in plants involves converting UDP-glucose into trehalose-6P using *TPS* and then converting *TPS* to trehalose using *TPP* (Avonce et al., 2006). Trehalose is also an osmoprotectant that prevents cell dehydration, stabilizes macromolecules, scavenges ROS, and improves cellular antioxidant properties (Raza et al., 2023). Applying a small quantity of trehalose can mitigate the detrimental effects of extreme temperatures on plants, enhance growth and development, and positively enhance plant resistance (Luo et al., 2018, 2022a). The heightened expression of sucrose and trehalose-related genes observed in this study notably augmented sucrose and trehalose levels (**Figure 7C**; **Supplementary Figure 8**). Prior research has demonstrated that trehalose up-regulates stress response genes, prompting the accumulation of osmolytes like proline, betaine, and soluble sugars, thereby influencing stress tolerance (Hassan et al., 2023). Moreover, heat stress significantly elevated proline levels in the root and shoot (**Figure 1B**; **Supplementary Table 7**). Soluble sugars can also reduce membrane osmotic potential and maintain cell expansion, possibly explaining why higher levels of sugars like glucose, sucrose, and trehalose can improve plant tolerance to abiotic stress.

**Figure 8:**
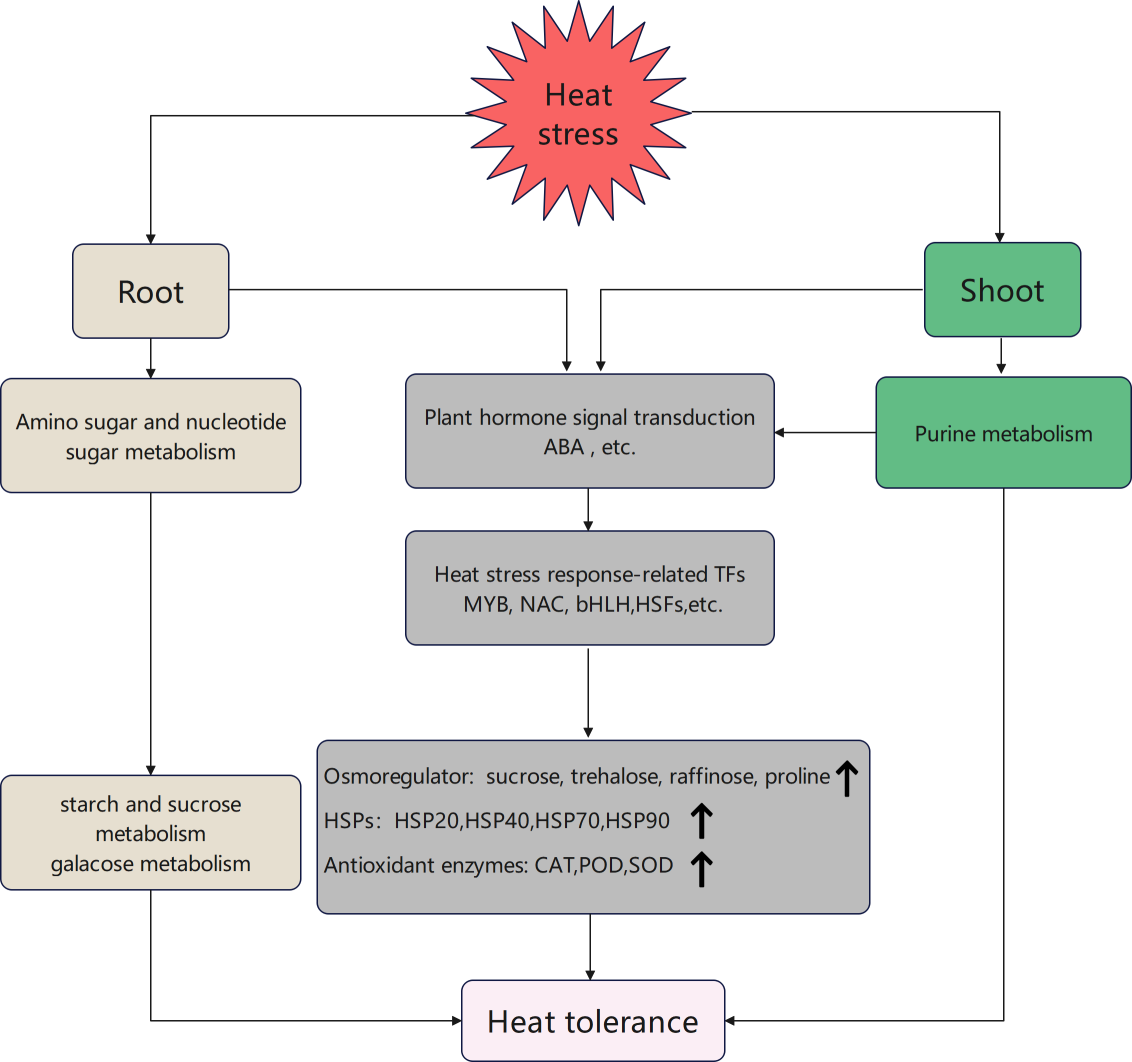
Model of heat stress response pathways in the root and shoot of tobacco seedlings.

Collectively, heat stress-related transcription factors (MYB, NAC, bHLH, and HSF) can regulate response to heat stress in the root and shoot through the plant hormone signal transduction pathway, which regulates HSP expression. Heat shock proteins can interact with osmotic regulators (trehalose and proline) and antioxidant enzymes (CAT, POD, and SOD) to regulate heat stress response. The root can engage in the pathways for sugar metabolism via the amino sugar and nucleotide sugar metabolism pathway to enhance raffinose, sucrose, and trehalose contents. The shoot can partake in synthesizing and signaling the phytohormone ABA through purine metabolism. This process can regulate sugar accumulation and ultimately enhance the tolerance of tobacco seedlings to high heat stress (**Figure 8**).

## Conclusion

This study integrated physiological and biochemical analyses with transcriptomic and metabolomic approaches to examine the molecular pathways involved in the response of tobacco seedling roots and shoots to heat stress. The primary findings of the study are outlined below. Heat stress significantly elevated the activities of antioxidant enzymes, including CAT, POD, and SOD in the shoot. Additionally, heat stress significantly increased the levels of osmotic mediators, such as soluble sugars and proline. Amino and nucleotide sugar metabolism were the primary metabolic pathways enriched in the root of heat-stressed tobacco, while purine metabolism was the predominant pathway in the shoot. These pathways can collectively enhance sugar metabolism, increasing the levels of sucrose, trehalose, raffinose, and other compounds that improve tolerance to heat stress in tobacco. While we have outlined the impact of the shoot on ABA levels via purine metabolism, leading to the regulation of HSP proteins and osmoregulatory substances through MYB and HSF transcription factors, as well as identified the positive role of the MYB transcription factor *NtMYB78* in heat stress regulation, the specific regulatory mechanisms remain unclear. Further validation of these molecular functions is required.

## Data availability statement

The original contributions presented in the study are publicly available. This data can be found here: https://www.ncbi.nlm.nih.gov/, the project number is PRJNA1093408.

## Author contributions

HC: Writing – review & editing, Writing – original draft. SQ: Writing – review & editing, Investigation, Methodology. YC: Resources, Validation, Writing – review & editing. JL: Writing – review & editing. TX: Resources, Writing – review & editing. PZ: Resources, Writing – review & editing. XS: Writing – review & editing. SX: Resources, Writing – review & editing. ZM: Resources, Writing – review & editing. ZH: Supervision, Writing – review & editing. XP: Writing – review & editing.

## Supplementary material

Supplementary Figure 1 Principle component analysis (PCA) of the transcriptome data. **(A)** The correlation heatmap of the 12 samples. R0 and S0 denote the root and shoot samples subjected to control conditions (25°C, 24 h), while R24 and S24 denote the samples subjected to heat stress (45°C, 24 h). The numbers 1, 2, and 3 correspond to the three biological replicates. **(B)** Principle components of differentially expressed genes (DEGs) in the root and shoot before and after heat treatment.

Supplementary Figure 2 Identification of DEGs. Root **(A)** and shoot **(B)** volcano plots of DEGs after heat treatments (before and after 24h). Red dots denote up-regulated genes, green dots denote down-regulated genes, and blue dots denote genes that were not differentially expressed. **(C)** The Venn diagram illustrates the up-regulated genes between the root and shoot tissues **(D)**. The Venn diagram illustrates the down-regulated genes in roots versus shoots.

Supplementary Figure 3 Relative expression levels of VIGS-mediated knock-down of gene *NtMYB78*.

Supplementary Figure 4 Expression of heat shock proteins (HSPs). Heat map of HSPs expression in the root **(A)** and shoot **(B)** before and after heat treatment with three biological replicates per treatment. **(C)** qRT-PCR validated the expression of *HSP20* (2), *HSP40* (2), *HSP70* (2) and *HSP90* (2). The expression level was normalized to that of *NtACTIN*. Data are given as means ± SD of three biological replicates. A significance analysis was performed using the Student’s t-test. **P* < 0.05, ****P* < 0.001.

Supplementary Figure 5 Distribution of identified metabolite species and PCA analysis. **(A, B)** show the classification and percentage of metabolites identified in the roots and shoots. PCA plots of total detected metabolites of R24 and R0 **(C)**, S24 and S0 **(D)**.

Supplementary Figure 6 Differential metabolite veen analysis. **(A, B)** indicates the veen graph that up-regulated DAMs and down-regulated DAMs in the root and shoot, respectively. **(C)** Heatmap of co-up-regulated DAMs in the root and shoot.

Supplementary Figure 7 Network diagram of key metabolic pathway genes and metabolite correlation. **(A)** The network diagram illustrates the correlations between genes and metabolites within the root-specific amino sugar and nucleotide sugar metabolism pathway that has been significantly enriched. **(B)** The network diagram illustrates the gene-metabolite correlations within the shoot-specific significantly enriched purine metabolic pathway. Green circles represent metabolites, while pink circles represent genes. Red lines denote positive correlations, while blue lines denote negative correlations.

Supplementary Figure 8 A network diagram of key metabolic genes and metabolite correlation. **(A)** A network of gene-metabolite correlations in the root and shoot featuring the significantly enriched galactose metabolism pathway. **(B)** A network of gene-metabolite correlations in the root and shoot featuring the significantly enriched starch and sugar metabolism pathway. Green circles indicate metabolites. Pink circles indicate genes. The red line indicates a positive correlation, and the blue line indicates a negative correlation. **(C)** Validation of *RS*, *SUS*, *TPS*, and *TPP* expression. Data was given as means ± SD of three biological replicates. A significance analysis was performed using the Student’s t-test. ***P* < 0.01, ****P* < 0.001.

Supplementary Figure 9 Heat map displaying the expression of ABA synthesis genes **(A)** and signaling genes **(B)**, with log_2_FC values representing alterations in gene expression. **(C)** The relative expression of *NCED1* in tobacco root and shoot. **(D)** The relative ABA content in the shoot.
