# Supplementary figures and images for: Integrated transcriptomics and metabolomics provides insights into the Nicotiana tabacum response to heat stress
Source: Front Plant Sci. 2024 Jul 22;15:1425944. doi: 10.3389/fpls.2024.1425944 (PMC11301762; doi:10.3389/fpls.2024.1425944)

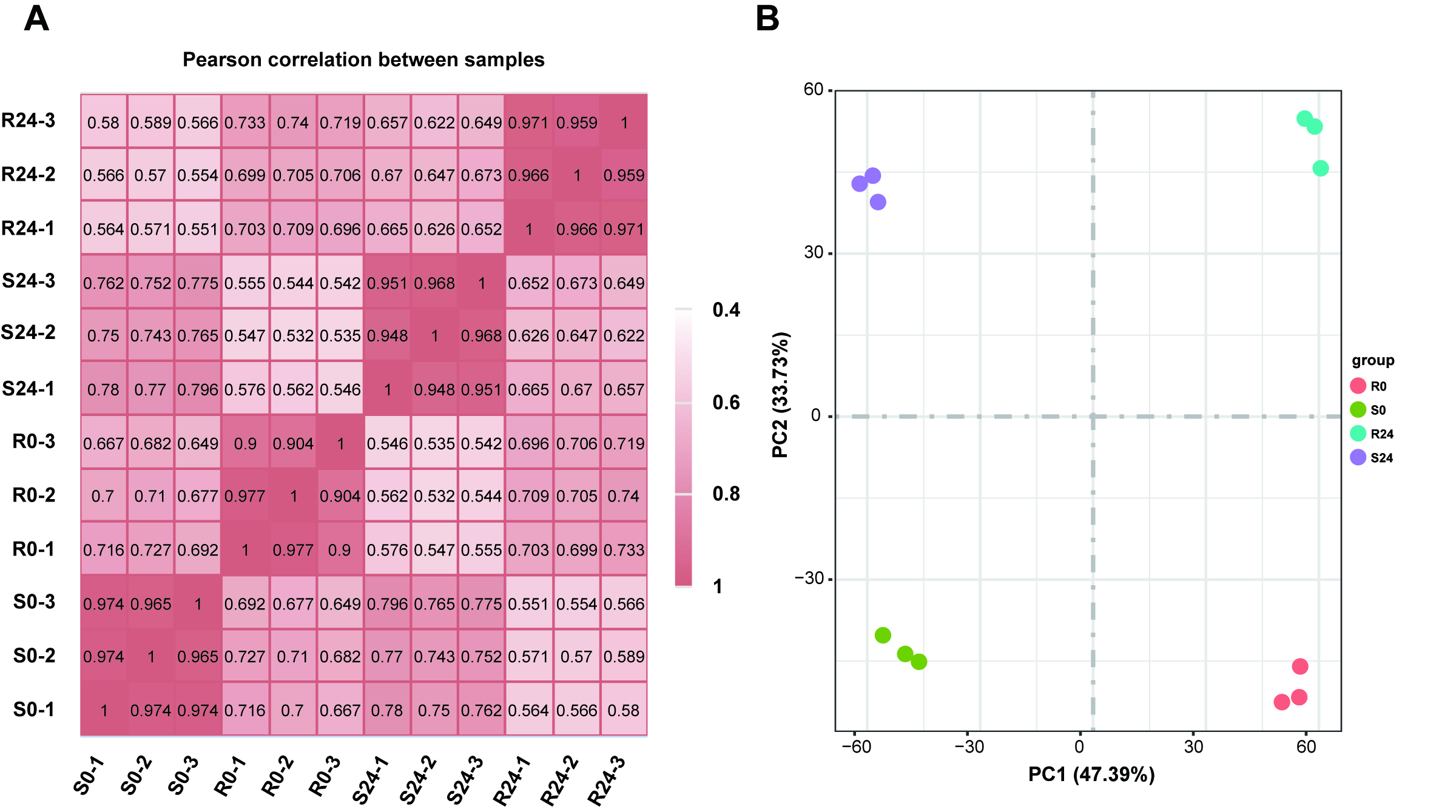

Supplement: Supplementary Figure 1 — Principle component analysis (PCA) of the transcriptome data. (A) The correlation heatmap of the 12 samples. R0 and S0 denote the root and shoot samples subjected to control conditions (25°C, 24 h), while R24 and S24 denote the samples subjected to heat stress (45°C, 24 h). The numbers 1, 2, and 3 correspond to the three biological replicates. (B) Principle components of differentially expressed genes (DEGs) in the root and shoot before and after heat treatment. [file Image_1.tif]

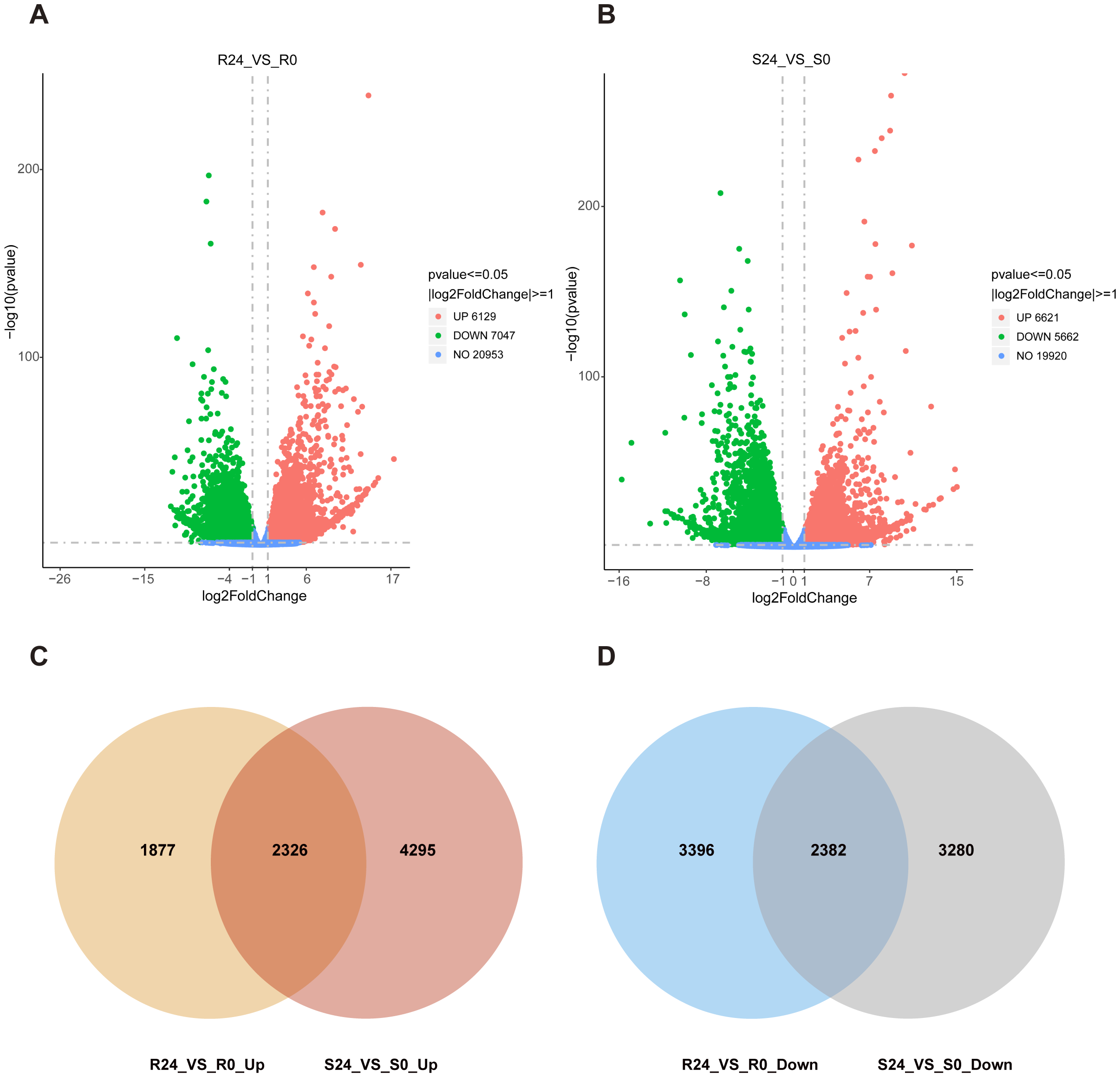

Supplement: Supplementary Figure 2 — Identification of DEGs. Root (A) and shoot (B) volcano plots of DEGs after heat treatments (before and after 24h). Red dots denote up-regulated genes, green dots denote down-regulated genes, and blue dots denote genes that were not differentially expressed. (C) The Venn diagram illustrates the up-regulated genes between the root and shoot tissues (D). The Venn diagram illustrates the down-regulated genes in roots versus shoots. [file Image_2.tif]

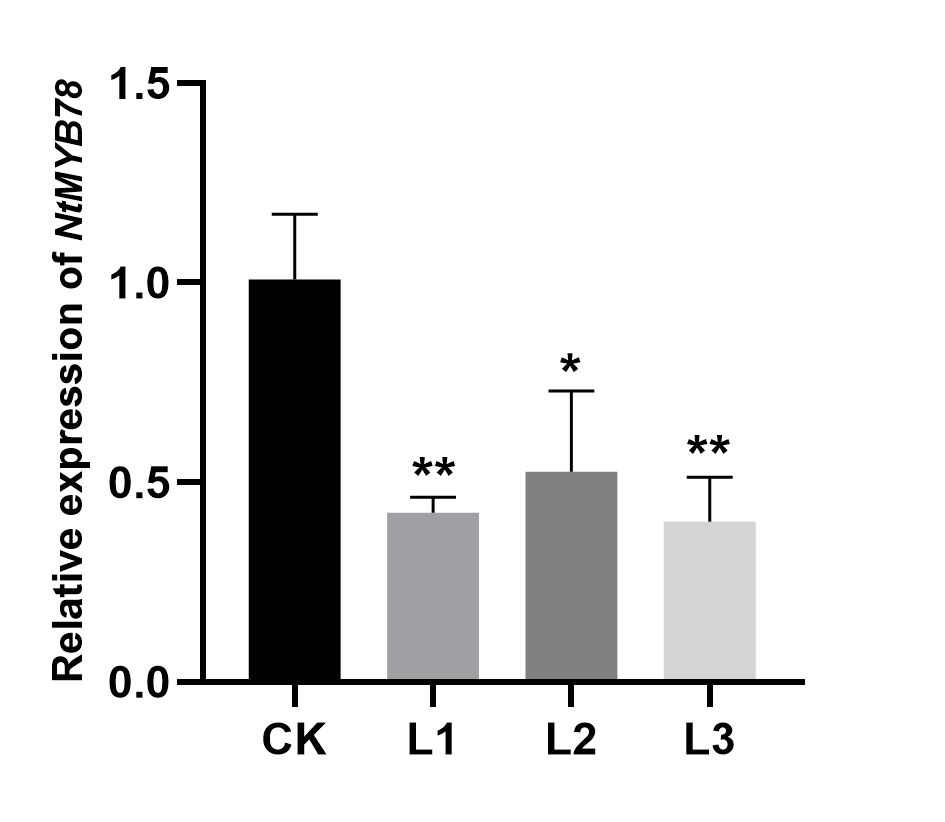

Supplement: Supplementary Figure 3 — Relative expression levels of VIGS-mediated knock-down of gene NtMYB78. [file Image_3.tif]

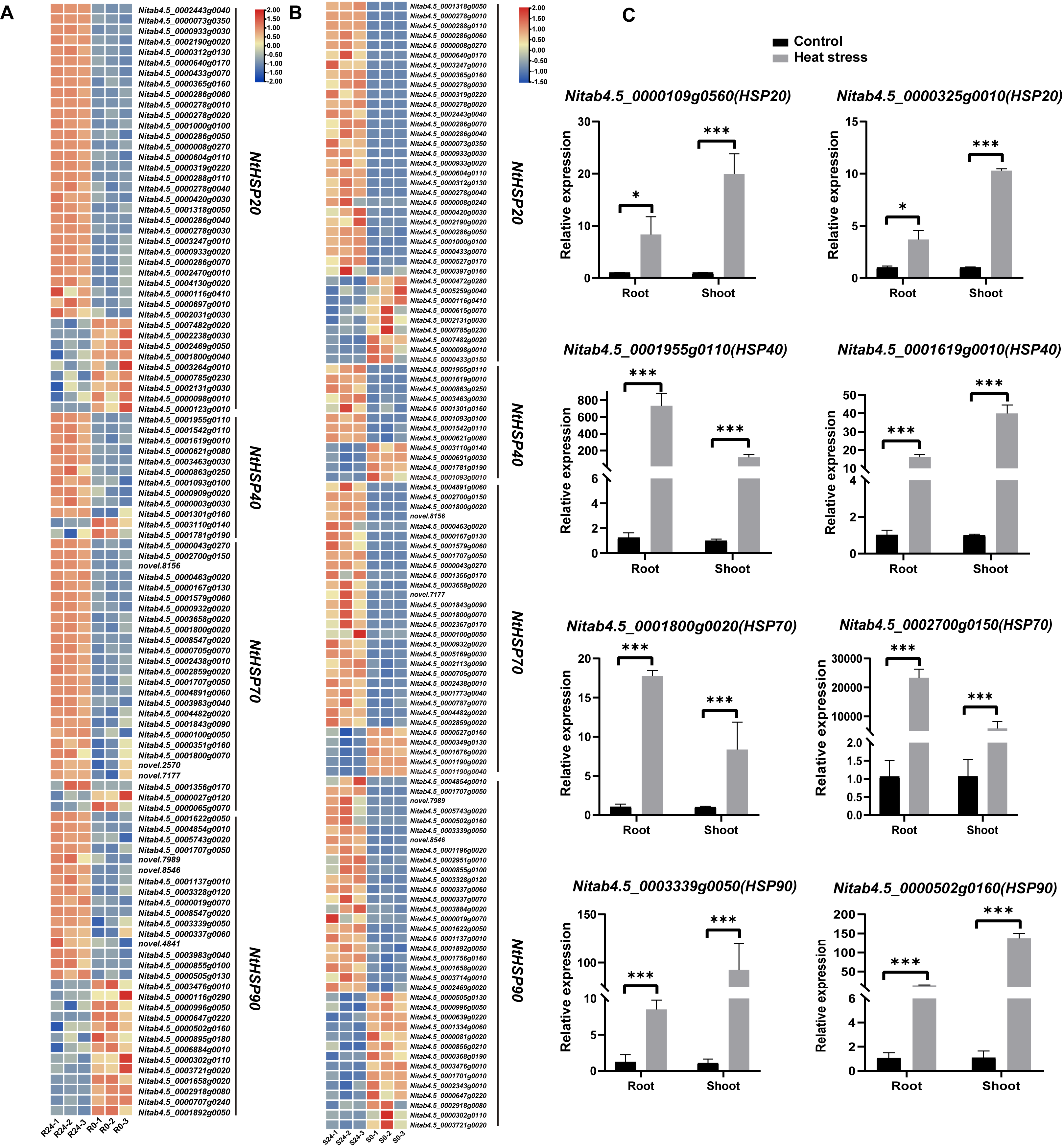

Supplement: Supplementary Figure 4 — Expression of heat shock proteins (HSPs). Heat map of HSPs expression in the root (A) and shoot (B) before and after heat treatment with three biological replicates per treatment. (C) qRT-PCR validated the expression of HSP20 (2), HSP40 (2), HSP70 (2) and HSP90 (2). The expression level was normalized to that of NtACTIN. Data are given as means ± SD of three biological replicates. A significance analysis was performed using the Student’s t-test. *P < 0.05, ***P < 0.001. [file Image_4.tif]

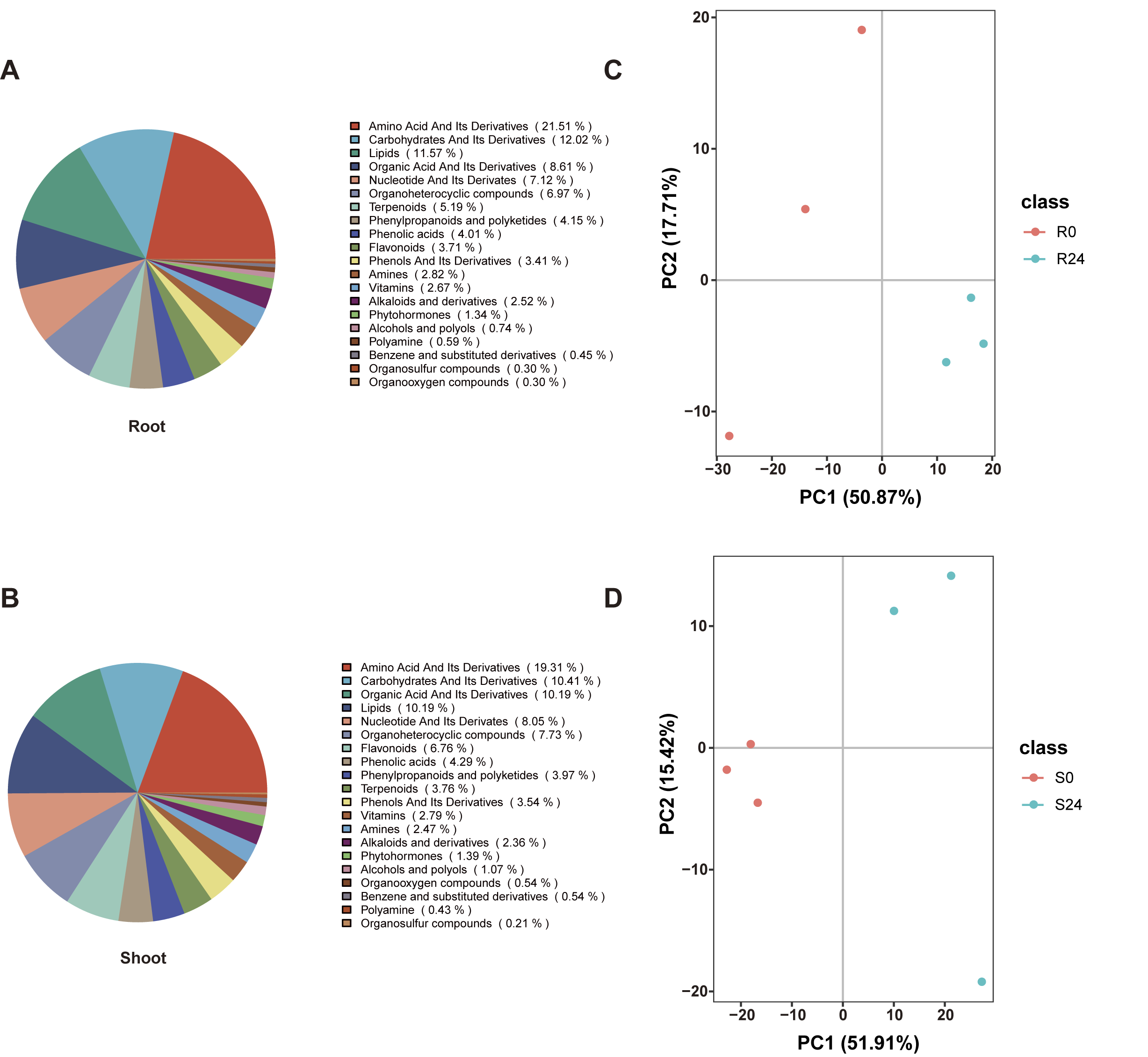

Supplement: Supplementary Figure 5 — Distribution of identified metabolite species and PCA analysis. (A, B) show the classification and percentage of metabolites identified in the roots and shoots. PCA plots of total detected metabolites of R24 and R0 (C), S24 and S0 (D). [file Image_5.tif]

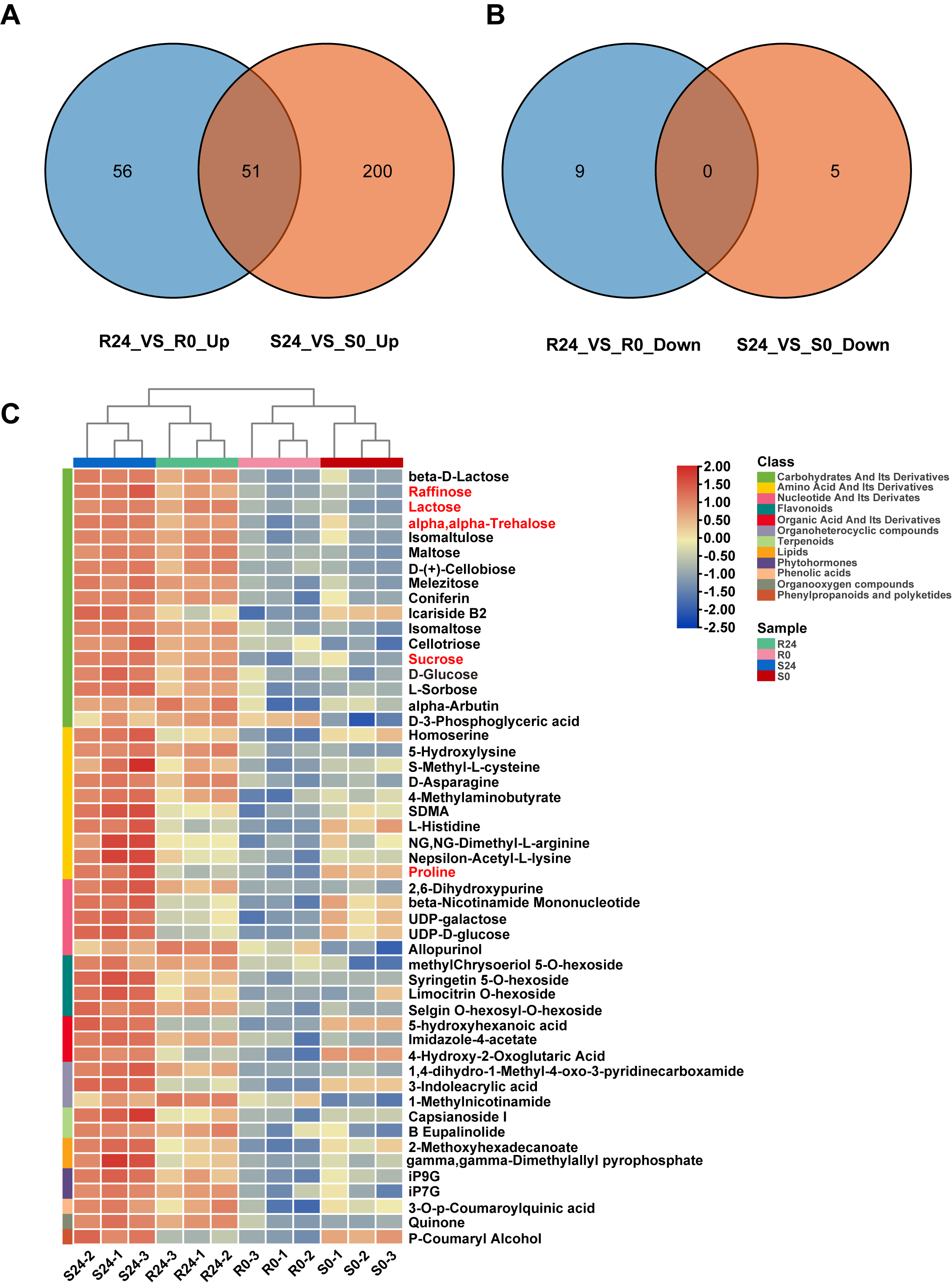

Supplement: Supplementary Figure 6 — Differential metabolite veen analysis. (A, B) indicates the veen graph that up-regulated DAMs and down-regulated DAMs in the root and shoot, respectively. (C) Heatmap of co-up-regulated DAMs in the root and shoot. [file Image_6.tif]

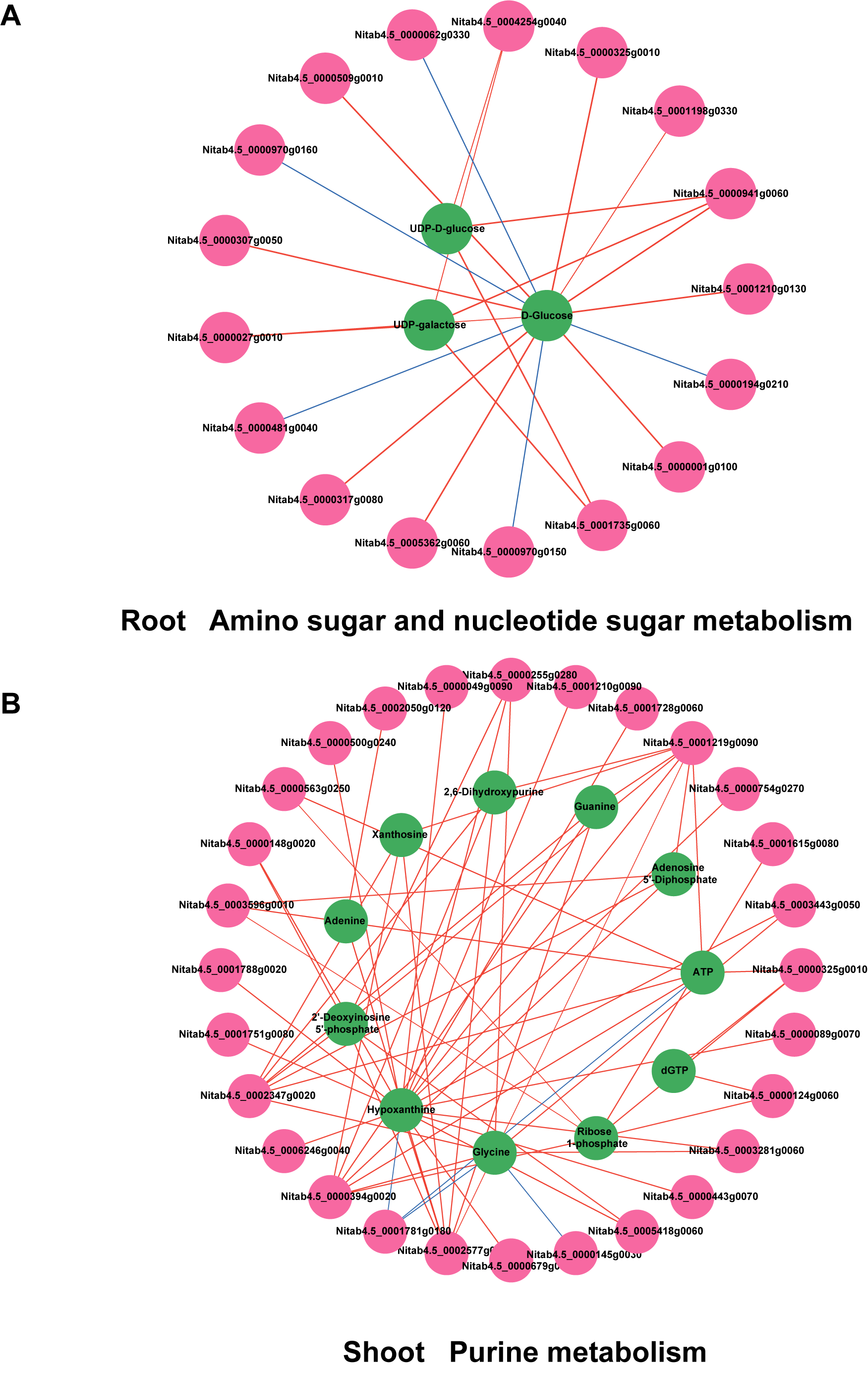

Supplement: Supplementary Figure 7 — Network diagram of key metabolic pathway genes and metabolite correlation. (A) The network diagram illustrates the correlations between genes and metabolites within the root-specific amino sugar and nucleotide sugar metabolism pathway that has been significantly enriched. (B) The network diagram illustrates the gene-metabolite correlations within the shoot-specific significantly enriched purine metabolic pathway. Green circles represent metabolites, while pink circles represent genes. Red lines denote positive correlations, while blue lines denote negative correlations. [file Image_7.tif]

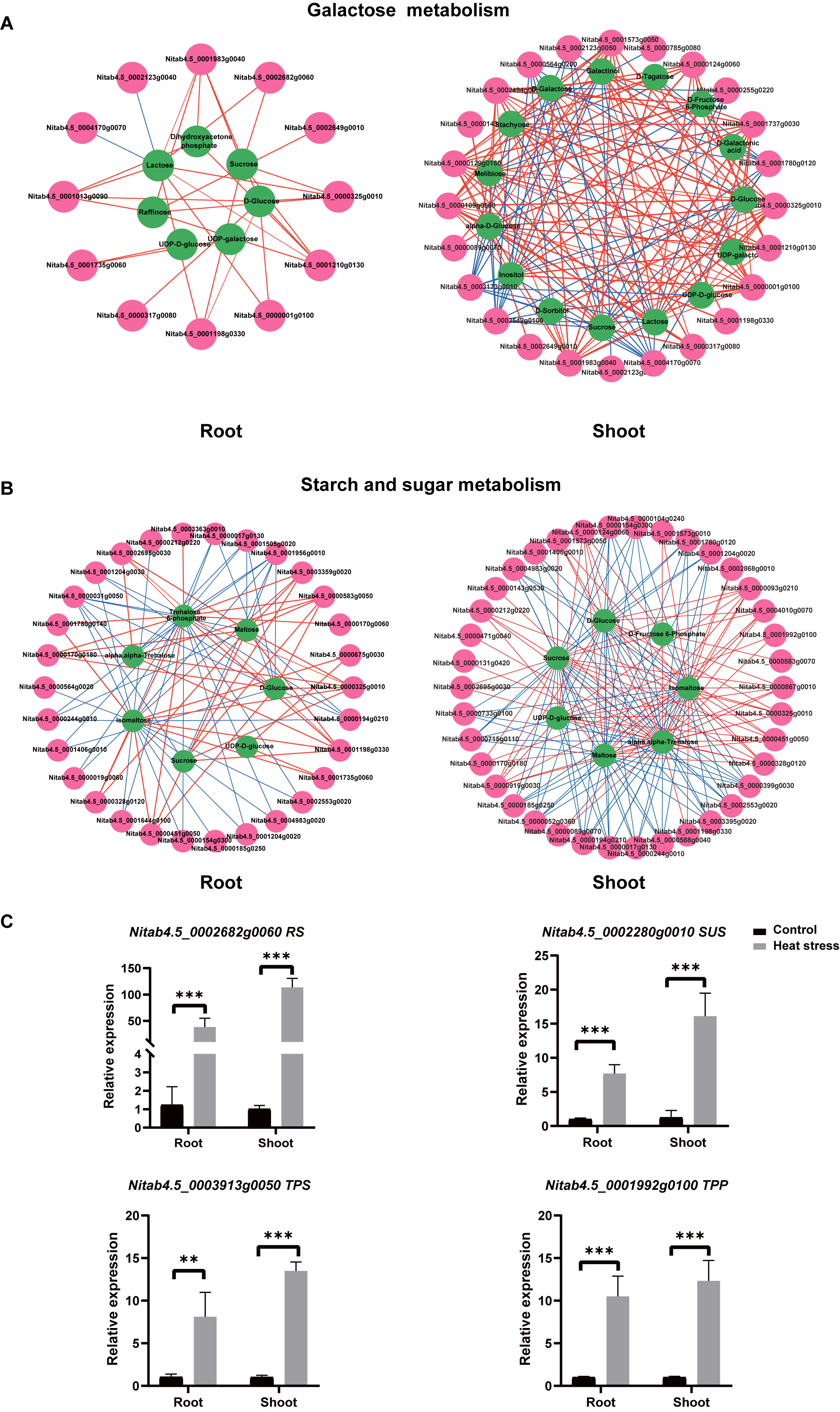

Supplement: Supplementary Figure 8 — A network diagram of key metabolic genes and metabolite correlation. (A) A network of gene-metabolite correlations in the root and shoot featuring the significantly enriched galactose metabolism pathway. (B) A network of gene-metabolite correlations in the root and shoot featuring the significantly enriched starch and sugar metabolism pathway. Green circles indicate metabolites. Pink circles indicate genes. The red line indicates a positive correlation, and the blue line indicates a negative correlation. (C) Validation of RS, SUS, TPS, and TPP expression. Data was given as means ± SD of three biological replicates. A significance analysis was performed using the Student’s t-test. **P < 0.01, ***P < 0.001. [file Image_8.tif]

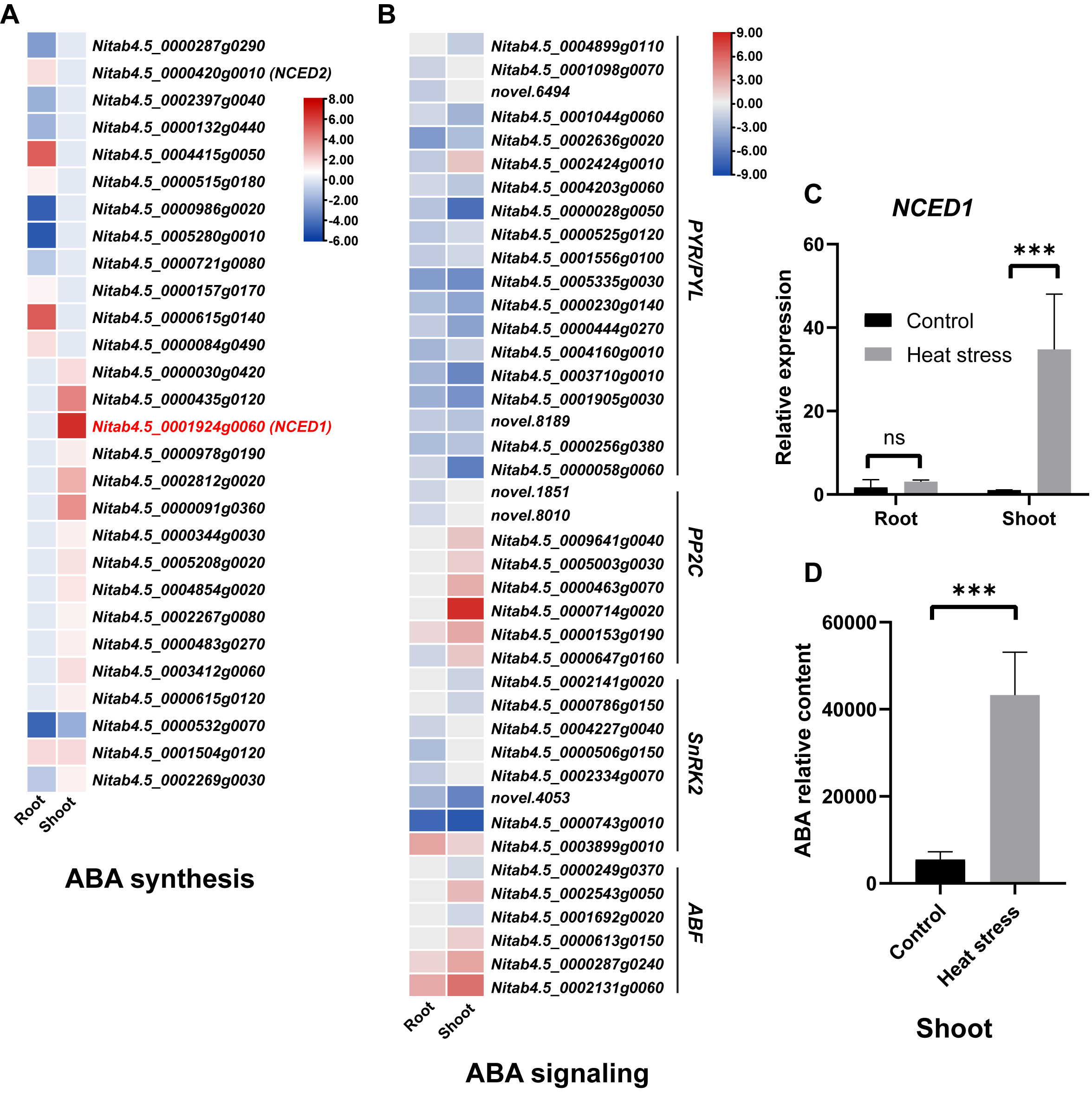

Supplement: Supplementary Figure 9 — Heat map displaying the expression of ABA synthesis genes (A) and signaling genes (B), with log2FC values representing alterations in gene expression. (C) The relative expression of NCED1 in tobacco root and shoot. (D) The relative ABA content in the shoot. [file Image_9.tif]
